# Developmental Dimensions in Preterm Infants During the 1st Year of Life: The Influence of Severity of Prematurity and Maternal Generalized Anxiety

**DOI:** 10.3389/fpsyg.2020.00455

**Published:** 2020-03-27

**Authors:** Erica Neri, Federica Genova, Fiorella Monti, Elena Trombini, Augusto Biasini, Marcello Stella, Francesca Agostini

**Affiliations:** ^1^Department of Psychology, University of Bologna, Bologna, Italy; ^2^Donor Human Milk Bank Italian Association (AIBLUD), Milan, Italy; ^3^Paediatric and Neonatal Intensive Care Unit, Maurizio Bufalini Hospital, Cesena, Italy

**Keywords:** infant outcome, maternal anxiety, extremely low birth weight, very low birth weight, trajectories of development

## Abstract

**Background:**

The literature has recognized premature birth as a risk factor for infant development and maternal anxiety. This study investigated the impact of the severity of birth weight, as well as of maternal anxiety at 3 months of infants’ corrected age, on infants’ outcomes during the 1st year postpartum. Moreover, it described the longitudinal trajectories of developmental outcomes, additionally exploring the impact of anxiety.

**Methods:**

The study compared 147 mothers and their 147 newborns, differentiated in 25 Extremely Low Birth Weight (ELBW), 41 Very Low Birth Weight (VLBW), and 81 Full-Term (FT) infants. At 3, 9, and 12 months (corrected age in the case of preterm infants) the level of infants’ development was investigated according to the 5 quotients (Locomotor, Personal and Social, Hearing and Language, Eye-hand Co-ordination and Performance) of the Griffiths Mental Development Scales (GMDS-R). During the assessment of 3 months, mothers fulfilled Penn State Worry Questionnaire (PSWQ) to evaluate the presence of generalized anxiety.

**Results:**

Among the 5 GMDS-R quotients, significant effect of severity of birth weight emerged only for Performance quotient: preterm infants (ELBW at 3 months; VLBW at 12 months) showed lower scores than FT ones. Moreover, this quotient decreased from 3 to 9 and to 12 months for VLBW and FT infants, while it was stable for ELBW ones. A significant interaction between severity of birth weight and maternal anxiety emerged for Hearing and Language and Locomotor quotients. In the first case, scores for ELBW infants, independently from maternal anxiety, decreased from 9 to 12 months. The same results emerged for VLBW infants, in the case of non-anxious mothers. Regarding Locomotor quotient, mean scores decreased from 3 to 9 and to 12 months for all groups in the case of non-anxious mothers. Conversely, when mothers were anxious, this decrease emerged only for VLBW infants. Lastly, ELBW, VLBW and FT showed difference in the growth and slope of the trajectories of different quotients.

**Conclusion:**

The severity of birth weight for preterm infants, also in interaction with maternal anxiety, had significant and specific impact on different dimensions of infants’ development. Clinical implications of these results underline the need for individualized interventions.

## Introduction

Prematurity is an unexpected and traumatic event during childbirth (Korja et al., 2012; Helle et al., 2016; Neri et al., 2017) and it represents a serious risk factor for child development, with possible sequelae and/or impairments in the brief and long term (Anderson, 2014; Jarjour, 2015; Rogers and Hintz, 2016; Vungarala and Rajeswari, 2018; Marchman et al., 2019).

A preterm birth also negatively influences the transition to parenthood (Rieves et al., 2016; Kerr et al., 2017), as parents, especially mothers, feel disoriented and frightened and might experience feelings of guilt, grief and recurrent worries about their baby’s survival and health (Mendelsohn, 2005; Korja et al., 2009, 2010; Shah et al., 2011; Lasiuk et al., 2013; Ionio et al., 2019; Pisoni et al., 2019).

According to an estimation by the World Health Organization, each year approximately 15 million babies are born prematurely, worldwide (Blencowe et al., 2012). In Italy, preterm birth occurs in most out of 4000 labors, with a rate of 7–8% (World Health Organization, 2012; Delnord et al., 2017; Granese et al., 2019).

Prematurity is globally defined as every childbirth which occurs before the 37 gestational weeks (World Health Organization, 2012); today, preterm infants represent a large and heterogeneous population according to their clinical conditions. Indeed, in the last decades, medical and technological advances have allowed the survival of babies who are ever smaller for gestational age and birth weight (World Health Organization, 2012; Lee et al., 2019). In particular, scientific literature actually distinguishes between “low-risk” preterm babies, with a birth weight between 1500 and 2500 grams (*Low Birth Weight*-LBW) and “high-risk” preterm infants, with birth weight less than 1500 grams (*Very Low Birth Weight*-VLBW), and specifically less than 1000 grams (*Extremely Low Birth Weight*-ELBW) (Lind et al., 2011; Biasini et al., 2012; Blencowe et al., 2012; Mariani et al., 2018).

The risk of sequelae, including neurodevelopmental delays, is inversely proportional to infant birth weight (Johnson, 2007; Lind et al., 2011; Biasini et al., 2012; Neri et al., 2017) and is significantly higher in populations of ELBW (Johnson et al., 2009; Rogers and Hintz, 2016) and VLBW (Murray et al., 2014), when compared to groups of full-term infants.

Recent studies and reviews on ELBW or VLBW sequelae report evidence regarding different developmental domains, such as: neurosensory (Marlow et al., 2005; Doyle et al., 2010), motor (Williams et al., 2010; Van Hus et al., 2014; Hughes et al., 2016), linguistic (Reidy et al., 2013; Guarini et al., 2016; Vandormael et al., 2019), personal-social (Montagna and Nosarti, 2016; Caldas et al., 2018), and cognitive (Bhutta et al., 2002; Kerr-Wilson et al., 2012; Sansavini et al., 2015; Stålnacke et al., 2019). However, previous research typically focused on specific and selected areas only, with very few studies considering multiple dimensions in unison (Duncan et al., 2012; Greene et al., 2013; Lobo et al., 2014), finding high prevalence of delays especially in cognitive, language and motor development.

Furthermore, most studies have often focused on one specific population (VLBW or ELBW), neglecting the comparison between the 2 groups. It is reasonable to speculate that ELBW and VLBW infants may show specific and somewhat different profiles concerning impairments, needs and resources, as we have already discovered in previous studies (Agostini et al., 2014; Neri et al., 2015, 2017). Specifically, we observed that preterm infants’ outcomes were worse in the group of ELBW when compared to VLBW and full-term ones. Furthermore, The latter groups showed similar performance in most of the domains investigated (Neri et al., 2017). If, on one hand, it is evident that preterm birth makes infants more vulnerable to a generalized delay in the development, on the other hand, the quality of preterm developmental outcomes may vary greatly, including both fragile and adaptive areas simultaneously. The focus on specific developmental dimensions, therefore, may provide important information for the impaired domains and potential resources in specific preterm populations.

In fact, due to the influence of multiple and heterogeneous variables, the trajectories of preterm infant development may show a wide range of variability from child to child.

Other than considering the role played by the biological and neurodevelopmental factors, we have to include environmental variables, which may interfere with/or positively influence preterm baby growth. For example, we can acknowledge the quality of the care provided by both the hospital environment and the staff, as well as the way in which the preterm baby’s parents react to the unexpected birth of their infant.

Indeed, many studies have, in the last years, focused on the investigation and description of emotional reactions and stress experienced by the mother after a premature childbirth. One of the most frequent consequences for maternal mental health is a heightened risk of experiencing different kinds of symptoms, such as traumatic stress symptomatology, depression, anxiety and acute stress disorder (Koutra et al., 2013; Rogers et al., 2013; Pace et al., 2016). In fact, preterm birth is a potential traumatic event for mothers, especially when the baby is VLBW or ELBW (Helle et al., 2018). Symptoms of depression and anxiety can also persist in parents (Pace et al., 2016) due to cumulative stress and daily challenges in learning the baby’s signals and how to reply to his/her needs sensitively.

Concerning the premature babies, the degree of severity of prematurity seems to be related to a high risk of maternal symptomatology. In fact, VLBW has been recognized as a relevant risk factor for preterm babies’ mothers, increasing the risk of being postnatally depressed from 4 to 18 times (Helle et al., 2015). Also, VLBW mothers showed a higher risk of developing acute stress disorder and high levels of posttraumatic stress symptoms (Helle et al., 2018).

Perinatal anxiety has been defined as anxiety experienced during the antenatal and/or postpartum period (first 12 months after birth) (Leach et al., 2017). Despite symptomatology being very common at this time, if untreated, maternal anxiety represents a risk factor for both the woman’s and the baby’s health (Kim et al., 2015). In particular, anxiety in the 1st months postpartum has been associated with infant difficulties in the development of social and communicative skills over the subsequent months of life (Assel et al., 2002; Reilly et al., 2006; Kingston et al., 2012; Kim et al., 2015) and, in some cases, these difficulties could persist 2 years after childbirth (Kim et al., 2015).

Postnatal anxiety, in association with the possible negative consequences of a preterm birth, may impact on the child development. Indeed, we may hypothesize that maternal anxiety is expected to be particularly intense in the first postpartum months, usually corresponding to the period of NICU stay. Some empirical evidence would support this; for example, Feeley et al. (2005) found that highly anxious mothers at 3 months were less sensitive during the interactions with their VLBW infants at 3 and 9 months. Zelkowitz et al. (2009) found that, during NICU stay, mothers with high anxiety at 3 months postpartum were then less sensitive in interaction with their preterm infants and were less supportive and responsive at 24 months postpartum. Also, Zelkowitz et al. (2011) reported that mothers’ postnatal anxiety during the baby’s hospitalization was a relevant predictor of poorer cognitive development and more internalizing symptoms in VLBW infants at 24 months; however, maternal anxiety was not an independent predictor of motor development at 24 months corrected age. In both studies, Zelkowitz et al. (2009, 2011) focused on maternal anxiety at 3 months postpartum using a measure for trait anxiety, instead of a measure of state anxiety. In fact, the authors stressed the fact that, while some state anxiety is expected in most of the parents of VLBW infants, a higher level would be expected in those parents with trait anxiety, with more possible implications for child development.

It is notable that a debate on the specificity and characterization of perinatal anxiety, with obvious implications on the tools to use for the assessment, has received a growing interest from the scientific literature in the last few years (Fairbrother et al., 2019). Based on the theoretical and clinical approach, some studies have demonstrated empirical findings to suggest that perinatal anxiety would be in part different from the anxiety that manifests in other periods of a person’s life.

This is the case, for example, with the studies on “pregnancy-specific anxiety” (Huizink et al., 2004, 2016), or on perinatal worries (Moran et al., 2015). The latter in particular have been recently investigated in a study by Goldfinger et al. (2019), aimed at describing the content of worries and assessing worry severity (using the Penn State Worry Questionnaire) and generalized anxiety. Results evidenced that some perinatal women with a pervasive and disturbing level of perinatal-themed worries could be underestimated due to a normal level of generalized anxiety.

To sum up, despite the evidence of the influence of postnatal anxiety on parent-infant-relationships and child outcomes, the literature still shows a lack in investigating this issue. Moreover, the above-mentioned studies on preterm birth and maternal anxiety did not compare different subpopulations of preterm infants. In a previous study (Neri et al., 2015), we analyzed the influence of maternal anxiety considering 3 samples based on the severity of prematurity: ELBW, VLBW and FT samples. Results showed that, even if anxiety was higher in ELBW mothers, they demonstrated discrete levels of sensitivity during the interaction with their babies, while FT mothers, when anxious, were less sensitive.

## Aims of the Study

The literature has developed knowledge and findings on macro areas of child development in the case of preterm infants, such as language, attention and motor skills. However, there still is a lack of investigation regarding specific developmental dimensions, considering the severity of prematurity and specific maternal symptoms, especially in a longitudinal perspective.

Based on this, we developed a longitudinal study considering 3 specific time points that represent significant steps (3, 9, and 12 months) for the progress of infant development during the 1st year of life. Three months represent an important step for the detection of the early skills of the baby (for example, infants start to use their hands more intentionally, to reach their mouths or objects). At 9 months, new skills are supposed to emerge, like crawling and joint attention. At the end of the 1st year of life (12 months), the infant’s autonomy may be observed by the development of deambulation and/or the occurrence of the first words.

The first aim of this study was to investigate the impact of severity of preterm birth on specific areas of infant development (Locomotor, Personal-Social, Hearing and Language, Eye-hand Co-Ordination, Performance), at 3, 9, and 12 months of age, corrected for preterm infants. We hypothesized that, according to a higher degree of severity of premature birth (that is the case of ELBW), infant development would be worse compared to VLBW and FT. According to previous literature, we supposed that in ELBW infants, but not in VLBW and FT ones, scores in all dimensions would significantly decrease across the 1st year.

Secondly, we investigated whether maternal anxiety, at 3 months of the infant’s corrected age, could influence infant development in the different dimensions considered. Specifically, we hypothesized lower quotient scores according to the presence of both low birth weight and maternal anxiety.

Thirdly, we aimed at giving a description, through growth trajectories, of the different areas of development in ELBW, VLBW and FT infants from 3 to 12 months, considering also the effect of maternal anxiety.

## Materials and Methods

### Participants and Procedure

This study was part of a wider longitudinal research aimed to assess the course of infants’ development from 3 to 24 months postpartum.

All mothers were recruited according to the following exclusion criteria: presence of previous or present psychiatric illness, lack of fluency in Italian, presence of infants’ chromosomal abnormalities, cerebral palsy, malformations, fetopathy, severe complications (leukomalacia, hydrocoefalus, intraventricular hemorrhage of III–IV grades, retinopathy of prematurity, broncho-pulmonary dysplasia). In the case of twin birth, only the first-born one was included.

At the end of the recruitment, our sample included 147 mothers and their 147 newborns.

The Preterm (PT) group, recruited at Neonatal Intensive Care Unit (NICU) of Bufalini hospital (Cesena, Italy), was composed by 66 mothers and their 66 preterm infants (44.9% of the infants’ sample), with a birth weight under 1500 g and gestational age < 32 weeks. This group was differentiated in two groups: 41 mothers and their 41 babies with weight between 1000 and 1500 g and gestational age < 32 weeks (27.9% of the infants’ sample) constituted the VLBW group; 25 mothers and their 25 babies with weight under 1000 g and gestational age < 28 weeks (17% of the infants’ sample) constituted the ELBW group.

The Full Term (FT) group, recruited at the antenatal classes held in Cesena (Italy) during the third trimester of pregnancy, was composed by 81 mothers and their 81 full term healthy infants (55.1% of the infants’ sample), that had a birth weight > 2500 g and gestational age > 36 weeks.

All the assessments took place at “Anna Martini” University Laboratory (Department of Psychology, Bologna) at 3 months (T1), at 9 months (T2), and at 12 months postpartum (T3) (corrected age for preterm infants). During all the assessments, the level of infant development was evaluated by a trained psychologist according to the 5 quotients of the Griffiths Mental Development Scales (GMDS-R; Griffith, 1996).

At T1, all mothers, after providing their written informed consent, were asked to complete an *ad hoc* questionnaire regarding socio-demographic variables (age, education, marital status, parity) and infant information (birth weight, gestational age, gender, type of delivery, days of hospitalization). They were also asked to complete a self-report questionnaire aimed to assess the level of anxiety, while a trained psychologist assessed their infants’ development.

The Ethical Committee of the Department of Psychology (University of Bologna) approved the design of the study.

### Measures

The Griffiths Mental Development Scales (GMDS-R-Griffith, 1996) is a well-recognized measure of infants’ mental and psychomotor development. The assessment focused on 5 specific areas of development: Locomotor (A) measures postural control, balance, as well as abilities ranging from standing to walking; Personal and Social (B) measures interpersonal skills in entering into a relationship, through observation and questions addressed to the parent; Hearing and Language (C) measures the ability to listen to sounds and to reproduce them through imitation; Eye-hand Co-ordination (D) measures visual-motor coordination, which is fundamental for the development of manipulative skills; Performance (E) measures skills in manipulation, speed of working and precision, as well as the ability to apply them in novel situations. GMDS-R provides a quotient for each area of development, and a General developmental Quotient (GQ), representing the mean score of the 5 quotients. The scores are standardized for an expected value of 100 with SD of 16 for all the subscales and 12 for the General Quotient. Infants that score below 84 are considered at risk of neurodevelopmental impairment. Many studies on GMDS-R reported their validity and reliability (Bowen et al., 1996; Griffith, 1996). In the Italian context, they are widely used in the clinical follow-up of the preterm infants (Agostini et al., 2014; Neri et al., 2015, 2017).

The presence of maternal anxiety was investigated by the Penn State Worry Questionnaire (PSWQ; Meyer et al., 1990). The PSWQ is a self-report questionnaire aimed at assessing generalized pathological worries, considering their frequency and their degree of excessiveness and uncontrollability. It was developed to evaluate the individual’s disposition or tendency to generally worry. This questionnaire, among others on anxiety during the perinatal period, has been chosen because its focus on worries may facilitate the identification, in our sample, of women with a higher tendency of being troubled or disturbed by perinatal-themed concerns.

The PSWQ is composed by 16 items, rated on a Likert scale between 1 (“Not at all typical of me”) to 5 (“Very typical of me”). Eleven items are positively worded (e.g., “Once I start to worry, I can’t stop”), while five items are negatively worded (e.g., “I never worry about anything”). The sum of all items provides a total score that ranges from 16 to 80, where the higher the value, the higher the levels of pathological worry. In the present study, we administered the Italian version of PSWQ that showed good internal consistency (0.85), suggesting a clinical cut-off score ≥ 57 to discriminate anxious from non-anxious subjects (Morani et al., 1999).

### Statistical Analyses

Statistical analyses were carried out using the IBM SPSS statistical package version 25.0.

To verify the homogeneity among ELBW, VLBW and FT dyads regarding of socio-demographic and clinical variables, we performed Pearson’s Chi Square Test and Univariate Anova.

For the first and second purposes, Repeated Measures Manova were used to investigate the influence of specific factors (”Birth weight,” “Maternal Anxiety at T1,” and “Time of assessment”), and of their interactions, on the 5 quotients of GMDS-R continuous scores at T1, T2, and T3.

For the third purpose, growth curve analysis was used to describe trajectories of each GMDS-R quotients from T1 to T3 in ELBW, VLBW, and FT babies as a function of time and maternal anxiety at T1. With three repeated measures (i.e., T1, T2, and T3) of outcome variables, analyses were limited to linear and quadratic models (Field, 2014). Therefore, we assessed two alternative sets of growth curve models for each GMDS-R quotient: (1) a linear model with a random intercept and random slopes, which reflects linear change over time; (2) a quadratic model with a random intercept and random slopes, which reflects change that takes on a “U” or inverted “U” shape. These models were centered at the month during which the first data was collected (i.e., at T1) and, therefore, represented babies’ initial scores.

Modeling took place in two steps. Model 1 was fit as an unconditional growth model, where only the intercept, linear slope, and curved slope were specified in order to determine the trajectories of each GMDS-R quotients in ELBW, VLBW and FT babies irrespective of maternal anxiety at T1. Model fit was evaluated using the −2 log likelihood difference test (−2LL).

Model 2 was fit as a conditional growth model for exploring the effect of maternal anxiety at T1 on trajectories of each GMDS-R quotients in the three birth weight groups.

Significant results were considered when *p*-values were lower than 0.05.

## Results

### Sociodemographic and Clinical Characteristics of the Participants

Preliminary analyses showed that the 3 birth weight groups of dyads were homogeneous in relation to all socio-demographic and clinical variables, except for parity (χ^2^_(__2__)_ = 18.11; *p* < 0.0001), level of education (χ^2^_(__2__)_ = 13.12; *p* < 0.0001), and anxiety (χ^2^_(__2__)_ = 6.36; *p* = 0.042). In particular, FT mothers, compared to VLBW and ELBW ones, were primiparous, had graduated and were non-anxious in a higher percentage (Table 1).

**TABLE 1:** Dyads’ characteristics according to birth weight.

	**ELBW (*N* = 25)**	**VLBW (*N* = 41)**	**FT (*N* = 81)**	**F/X^2^**	***p*-Value**
**Maternal variables**					
**Age**				0.634	0.532
Mean (SD) (in years)	33.88 ± 4.52	34.21 ± 5.67	33.18 ± 4.75		
Marital status, n (%)				2.68	0.261
Married	24 (96%)	41 (100%)	75 (93.8%)		
Other	1 (4%)	0 (0.0%)	5 (6.3%)		
**Education, n (%)**				5.50	0.005
Primary and secondary school	9 (33.3%)	7 (17.1%)	6 (7.4%)		
High school and University	16 (66.7%)	34 (82.9%)	75 (92.6%)		
**Parity, n (%)**				36.19	<0.0001
Nulliparous	19 (76%)	26 (63.4%)	60 (75%)		
Multiparous	6 (24%)	15 (36.6%)	20 (25%)		
**Type of delivery, n (%)**				82.95	<0.0001
Spontaneous	6 (25%)	9 (23.7%)	60 (75%)		
Cesarean section	18 (75%)	29 (76.3%)	20 (25%)		
**Twinning, n (%)**				42.37	<0.0001
Yes	4 (16%)	16 (39%)	2 (2.5%)		
Not	21 (84%)	25 (61%)	79 (97.5%)		
**Maternal anxiety at T_1_, n (%)**				6.36	0.042
Anxious	6 (24%)	5 (12.2%)	5 (6.2%)		
Non-Anxious	19 (76%)	36 (87.8%)	76 (93.8%)		
**Infant variables**					
**Birth weight**				1005.69	<0.0001
Mean (SD) (in grams)	821.80 ± 100.04	1281.17 ± 163.93	3561.32 ± 415.72		
**Gestational age**				1066.80	<0.0001
Mean (SD) (in weeks)	27.44 ± 2.16	29.73 ± 1.64	40.03 ± 1.02		
**Days of hospitalization**				325.30	<0.0001
Mean (SD)	61.28 ± 16.43	34.97 ± 16.10	2.07 ± 0.31		
**Small gestational age, n (%)**				4.46	0.039
Yes	15 (60%)	34 (82.9%)	//		
Not	10 (40%	7 (17.1%)	//		
**Gender, n (%)**				2.19	0.334
Male	12 (48%)	15 (36.6%)	40 (49.4%)		
Female	13 (52%)	26 (63.4%)	41 (50.6%)		

Moreover, results showed significant differences in type of delivery (χ^2^_(__2__)_ = 36.19; *p* < 0.0001), twinning (χ^2^_(__2__)_ = 28.60; *p* < 0.0001), gestational age (*F*_(__2_,_143__)_ = 1066.80; *p* < 0.0001), days of hospitalization (*F*_(__2_,_143__)_ = 325.30; *p* < 0.0001), and small gestational age (χ^2^_(__2__)_ = 4.26; p. 039). Specifically, in FT mothers, cesarean section delivery and twinning were less frequent, compared to VLBW and ELBW mothers (Table 1). The differences that emerged, such as those concerning gestational age, days of hospitalization and small gestational age, were coherent with group belonging based on different birth weight. Because these variables were strictly linked to preterm status, they were not included in subsequent analyses. On the contrary, because “parity” and “level of education” were significantly associated with infants’ GMDS-R quotients, they were included in subsequent statistical analyses.

### Birth Weight and Infants’ Quotients From 3 to 12 Months Postpartum

Table 2 summarizes GMDS quotients of ELBW, VLBW and FT infants at 3, 9, and 12 months.

**TABLE 2:** Griffiths Mental Development Scales infants’ quotients according to birth weight and time of assessment.

	**Birth weight**			**Birth weight × Time of assessment**										
		
	**ELBW *N* = 25**	**VLBW *N* = 41**	**FT *N* = 81**	**ELBW *N* = 25**			**VLBW *N* = 41**			**FT *N* = 81**			**F**	
							
				**T_1_**	**T_2_**	**T_3_**	**T_1_**	**T_2_**	**T_3_**	**T_1_**	**T_2_**	**T_3_**	**Birth weight**	**Birth weight × Time of assessment**
Locomotor (Quotient A)	99.01 ± 16.24; 50–132	103.03 ± 16.44; 50–150	106.45 ± 16.35; 50–139	109.16 ± 12.29; 83–132	93.37 ± 13.71; 56–118	94.49 ± 18.01; 50–121	118.61 ± 13.67; 90–1500	96.63 ± 13.12; 72–122	93.85 ± 14.60; 69–129	115.59 ± 11.27; 90–139	101.89 ± 14.48; 53–130	101.85 ± 15.83; 50–133	2.016	2.054
Personal and Social (Quotient B)	95.17 ± 15.15; 62–139	94.35 ± 13.24; 59–139	101.24 ± 11.95; 59–130	103.07 ± 12.09; 74–125	90.80 ± 17.58; 62–139	91.64 ± 12.12; 64–118	103.35 ± 10.97; 91–136	89.28 ± 13.74; 67–128	90.41 ± 11.70; 73–122	108.58 ± 9.51; 85–125	96.34 ± 13.33; 59–125	98.78 ± 10.07; 72–130	2.842	0.233
Hearing and Language (Quotient C)	105.96 ± 10.29; 87- 135	106.90 ± 10.51; 77–150	106.91 ± 10.67; 77–150	104.67 ± 11.13; 87–135	111.48^e^ ± 7.32; 95–126	101.74^e^ ± 10.80; 89–129	106.46 ± 10.96; 92–135	109.81 ± 7.56; 99–126	104.43 ± 11.13; 85–132	106.34 ± 10.34; 77–135	106.49 ± 9.33; 85–135	107.91 ± 12.26; 85–150	0.097	5.052*
Eye-hand Coordination (Quotient D)	103.01 ± 15.02; 65–149	103.37 ± 14.51; 65–149	107.50 ± 14.42; 65–143	113.31 ± 14.87; 65–149	100.00 ± 12.17; 70–118	95.70 ± 13.41; 73–122	111.14 ± 10.75; 91–149	100.84 ± 11.35; 70–129	98.14 ± 16.10; 68–138	110.32 ± 15.51; 65–142	104.89 ± 12.86; 67–143	107.28 ± 13.83; 68–138	1.235	2.380
Performance (Quotient E)	100.34^a^ ± 12.30; 76–127	102.55^a^ ± 14.77; 62–150	111.31^a^ ± 15.14; 67–150	102.31^b^ ± 13.30; 76–125	99.21 ± 11.64; 86–127	99.49 ± 11.53; 80–122	114.36^bd^ ± 9.76; 90–125	95.29^d^ ± 13.12; 62–121	98.02^cd^ ± 12.72; 72–127	120.51^bd^ ± 10.65; 97–139	103.74^cd^ ± 15.32; 67–150	109.70^cd^ ± 14.61; 84–150	6.413*	3.365*

Values are mean ± SD; range * < 0.05 ^*a*^*p* < 0.005 for *post hoc* test (FT > VLBW and ELBW) ^*b*^*p* < 0.005 for *post hoc* test (ELBW < VLBW and FT) ^*c*^*p* < 0.05 for *post hoc* test (VLBW < FT) ^*d*^*p* < 0.05 for *post hoc* test (T_1_ > T_2_ and T_3_) ^*e*^*p* < 0.0001 for *post hoc* test (T_2_ > at T_3_).

In line with the first aim, we explored the impact of birth weight, as well as of the interaction between birth weight and time of assessment, on infants’ GMDS-R quotients.

When the impact of birth weight was considered, results showed a significant effect on Performance quotient (*F*_(__2_,_130__)_ = 6.413; *p* = 0.002): FT infants had significantly higher mean score than those observed in VLBW and ELBW infants (Bonferroni *post hoc* test *p* < 0.0001 and *p* = 0.002, respectively) (Table 2).

**FIGURE 1:**
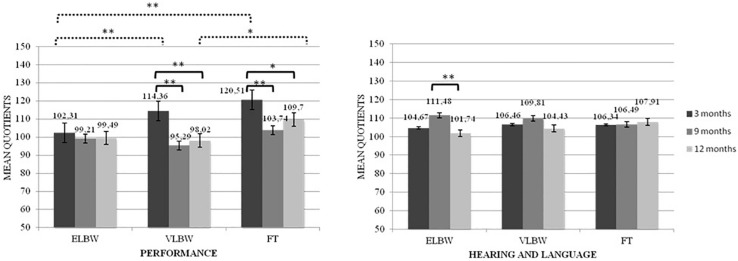
Performance and Healing and Language quotients according to the interaction between birth weight and time of assessment. *p < 05; **p < 0.005. Continue line denotes within group comparison; dotted line between group comparison.

When the interaction between birth weight and time of assessment was considered, results showed significant differences both between and within the 3 birth groups of infants on Performance quotient (*F*_(__2_,_130__)_ = 3.365; *p* = 0.038). Looking at the differences between groups, at T1, ELBW infants showed a significantly lower mean score than that reported by FT and VLBW infants (Bonferroni *post hoc* test *p* < 0.005 and *p* < 0.0001, respectively); while, at T3, VLBW infants showed a significantly lower mean score than that observed in FT infants (Bonferroni *post hoc* test *p* = 0.033). Looking at the differences within groups, mean scores of both FT and VLBW groups significantly decreased from T1 to T2 (Bonferroni *post hoc* test *p* < 0.005 and *p* < 0.0001, respectively) and from T1 to T3 (Bonferroni *post hoc* test *p* = 0.036 and *p* < 0.0001, respectively). No differences emerged in the ELBW group (Figure 1).

The interaction between birth weight and time of assessment also showed significant differences in the Hearing and Language quotient (*F*_(__2_,_130__)_ = 5.052; *p* = 0.008): ELBW infants showed a significantly higher mean score at T2 than that observed at T3 (Bonferroni *post hoc* test *p* < 0.0001) (Figure 1).

No significant differences emerged for the other GMDS-R quotients (Table 2).

Table 3 presents rates of delay (< 1 DS) of ELBW, VLBW and FT infants at 3, 9, and 12 months.

**TABLE 3:** Prevalence of delay (< 1 DS) on the GMDS-R.

		**Birth weight × Maternal anxiety × Time of assessment**								
		
		**ELBW**			**VLBW**			**FT**		
				
	**Maternal anxiety**	**T_1_**	**T_2_**	**T_3_**	**T_1_**	**T_2_**	**T_3_**	**T_1_**	**T_2_**	**T_3_**
Locomotor (Quotient A)	Total Sample	1 (4.0)	5 (20.0)	5 (20.0)	0 (0.0)	5 (12.2)	5 (12.2)	0 (0.0)	15 (19.2)	10 (13.0)
	Not anxious	1 (5.3)	4 (21.1)	4 (21.1)	0 (0.0)	5 (13.9)	3 (8.3)	0 (0.0)	15 (20.5)	10 (13.9)
	Anxious	0 (0.0)	1 (16.7)	1 (16.1)	0 (0.0)	0 (0.0)	2 (40.0)	0 (0.0)	0 (0.0)	0 (0.0)
Personal and Social (Quotient B)	Total Sample	1 (4.0)	11 (44.0)	4 (16.0)	0 (0.0)	14 (34.1)	6 (14.6)	0 (0.0)	12 (15.4)	3 (3.9)
	Not anxious	1 (5.3)	8 (42.1)	2 (10.5)	0 (0.0)	11 (30.6)	4 (11.1)	0 (0.0)	11 (15.1)	3 (4.2)
	Anxious	0 (0.0)	3 (50.0)	2 (33.3)	0 (0.0)	3 (60.0)	2 (40.0)	0 (0.0)	1 (20.0)	0 (0.0)
Hearing and Language (Quotient C)	Total Sample	0 (0.0)	0 (0.0)	0 (0.0)	0 (0.0)	0 (0.0)	0 (0.0)	2 (2.5)	0 (0.0)	0 (0.0)
	Not anxious	0 (0.0)	0 (0.0)	0 (0.0)	0 (0.0)	0 (0.0)	0 (0.0)	2 (1.2)	0 (0.0)	0 (0.0)
	Anxious	0 (0.0)	0 (0.0)	0 (0.0)	0 (0.0)	0 (0.0)	0 (0.0)	0 (0.0)	0 (0.0)	0 (0.0)
Eye-hand Co-ordination (Quotient D)	Total Sample	1 (4.0)	1 (4.0)	6 (24.0)	0 (0.0)	1 (2.4)	9 (22.0)	3 (3.7)	3 (3.8)	7 (9.1)
	Not anxious	1 (5.3)	1 (5.3)	6 (31.6)	0 (0.0)	1 (2.8)	8 (22.2)	3 (3.7)	3 (4.1)	7 (9.7)
	Anxious	0 (0.0)	0 (0.0)	0 (0.0)	0 (0.0)	0 (0.0)	1 (20.0)	0 (0.0)	0 (0.0)	0 (0.0)
Performance (Quotient E)	Total Sample	2 (8.0)	0 (0.0)	4 (16.0)	0 (0.0)	5 (12.2)	6 (14.6)	0 (0.0)	12 (15.4)	1 (1.3)
	Not anxious	1 (5.3)	0 (0.0)	3 (15.8)	0 (0.0)	4 (11.1)	5 (13.9)	0 (0.0)	11 (15.1)	1 (1.4)
	Anxious	1 (5.3)	0 (0.0)	1 (16.7)	0 (0.0)	1 (20.0)	1 (20.0)	0 (0.0)	1 (20.0)	0 (0.0)

The majority of VLBW and FT infants did not show delays at T1, while a small percentage was observable at T2 and T3. Conversely, ELBW showed delays in all three assessments.

When specific quotients are observed, a low number of cases emerge in Hearing and Language scores, while a high rate emerges in Locomotor ones.

### Birth Weight and Maternal Anxiety on Infants’ Quotients From 3 to 12 Months Postpartum

In line with the second aim, we investigated the interaction between birth weight, maternal anxiety and time of assessment on infants’ GMDS-R quotients. All results are shown in Table 4.

**TABLE 4:** Griffiths Mental Development Scales infants’ quotients according to the interaction between birth weight.

		**Birth weight × Maternal anxiety × Time of assessment**									
		
		**ELBW**			**VLBW**			**FT**			**F**
					
	**Maternal Anxiety**	**T_1_**	**T_2_**	**T_3_**	**T_1_**	**T_2_**	**T_3_**	**T_1_**	**T_2_**	**T_3_**	**Birth weight × Maternal anxiety × Time of assessment**
Locomotor (Quotient A)	Not anxious	110.80^ad^ ± 13.00; 83–132	92.52^a^ ± 11.68; 79–118	92.77^a^ ± 16.33; 57–121	118.57^a^ ± 14.49; 90–150	102.44^a^ ± 13.64; 72–122	100.29^a^ ± 14.48; 69–129	119.78^ad^ ± 10.66; 97–139	98.92^a^ ± 14.53; 53–130	97.28^a^ ± 15.99; 50–133	3.274*
	Anxious	107.53 ± 10.53; 90–118	94.22 ± 20.23; 56–115	96.21 ± 24.36; 50–117	118.64^a^ ± 5.58; 111–125	90.82^a^ ± 4.89; 87–99	87.40^a^ ± 10.03; 73–97	111.41 ± 18.78; 90–132	104.86 ± 13.86; 88–118	106.42 ± 10.80; 93–121	
Personal and Social (Quotient B)	Not anxious	105.02 ± 12.61; 74–125	89.03 ± 13.26; 62–123	92.52 ± 12.76; 64–118	107.48 ± 10.90; 91–136	94.13 ± 13.85; 67–128	93.92 ± 11.25; 73–122	108.39 ± 9.72; 85–125	97.83 ± 13.38; 59–125	99.38 ± 10.17; 72–130	0.090
	Anxious	101.13 ± 11.02; 85–119	92.57 ± 29.00; 62–139	90.76 ± 10.69; 81–110	99.22 ± 9.62; 91–114	84.44 ± 10.87; 73–100	86.91 ± 13.86; 73–110	108.78 ± 6.00; 102–114	94.86 ± 13.77; 78–115	98.19 ± 9.49; 85–110	
Hearing and Language (Quotient C)	Not anxious	105.49 ± 12.76; 87–135	109.07^b^ ± 5.14; 99–118	102.42^b^ ± 12.96; 89–129	107.33^c^ ± 10.76; 92–135	113.54^bcd^ ± 7.61; 99–126	104.78^b^ ± 11.59; 85–132	106.96 ± 10.51; 77–135	108.18^d^ ± 9.53; 85–135	105.01 ± 12.23; 85–150	3.255*
	Anxious	103.85 ± 8.36; 98–119	113.89^b^ ± 11.80; 95–126	101.06^b^ ± 5.00; 96–107	105.59 ± 13.54; 92–119	106.07 ± 4.09; 100–110	104.08 ± 7.78; 100–118	105.71 ± 8.04; 98–114	104.80 ± 5.21; 100–112	110.81 ± 11.18; 107–132	
Eye-hand Co-ordination (Quotient D)	Not anxious	109.98 ± 16.42; 65–149	98.99 ± 12.16; 70–188	93.93 ± 13.83; 73–122	112.35 ± 10.91; 91–149	102.67 ± 11.67; 70–129	98.84 ± 11.61; 68–138	110.43 ± 15.38; 65–142	101.40 ± 12.63; 67–143	104.15 ± 13.74; 68–138	0.258
	Anxious	116.65 ± 7.68; 104–126	101.01 ± 13.37; 86–118	97.48 ± 8.11; 89–111	109.92 ± 9.93; 96–119	99.01 ± 8.67; 86–107	97.45 ± 13.21; 79–114	110.23 ± 19.41; 88–134	108.38 ± 16.45; 95–138	110.41 ± 15.69; 90–133	
Performance (Quotient E)	Not anxious	93.93 ± 12.62; 76–125	98.72 ± 10.50; 86–121	98.77 ± 11.49; 80–122	111.77 ± 9.82; 90–125	98.50 ± 13.80; 62–121	100.92 ± 12.59; 72–127	118.50 ± 10.85; 97–139	101.54 ± 14.52; 67–150	109.61 ± 14.13; 84–150	2.791
	Anxious	97.48 ± 13.58; 76–11	99.70 ± 15.97; 86–127	100.22 ± 12.72; 80–114	116.95 ± 9.12; 104–125	92.09 ± 12.30; 74–103	95.12 ± 14.57; 104–125	122.51 ± 6.26; 118–132	105.93 ± 26.44; 83–150	109.79 ± 22.55; 97–150	

Maternal anxiety and time of assessment. Values are mean ± SD; range *p < 0.05 ^*a*^*p* < 0.005 for *post hoc* test (T_1_ > T_2_ and T_3_) ^*b*^*p* < 0.05 for *post hoc* test (T_2_ > T_3_) ^*c*^*p* < 0.05 for *post hoc* test (T_1_ < T_2_) ^*d*^*p* < 0.05 for *post hoc* test (FT > VLBW and ELBW.

Regarding the interaction between birth weight and maternal anxiety, no significant differences emerged.

When the interaction among birth weight, maternal anxiety and time of assessment was considered, significant results emerged on Locomotor (*F*_(__2_,_130__)_ = 3.274; *p* = 0.041) and Hearing and Language quotients (*F*_(__2_,_130__)_ = 3.255; *p* = 0.042).

**FIGURE 2:**
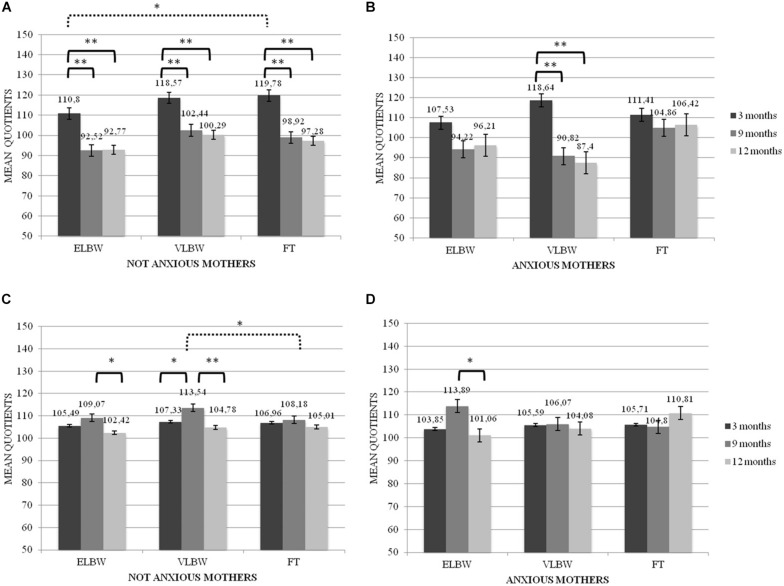
Locomotor and Hearing and Language quotients according to the interaction between birth weight, maternal anxiety and tune of assessment. *p < 05; **p < 0.005. **(A)** Locomotor mean Quotients in Not Anxious group, **(B)** Locomotor mean Quotients in Anxious group, **(C)** Hearing and Language mean Quotients in Not Anxious group, **(D)** Hearing and Language mean Quotients in Not Anxious group. Continue line denotes within group comparison, dotted line between group comparison.

Regarding the Locomotor quotient, results showed significant differences both between and within the 3 birth groups of infants. First, in the case of non-anxious mothers, at T1 FT infants had significantly higher mean score than that reported by ELBW ones (Bonferroni *post hoc* test *p* = 0.022) (Figure 2A). Looking at the group differences, in the case of non-anxious mothers, the mean scores observed at T1 were significantly higher than those observed at T2 and at T3 in ELBW, VLBW and FT infants (Bonferroni *post hoc* test *p* < 0.0001, respectively) (Figure 2A). In the case of anxious mothers, this effect emerged only for VLBW infants: at T1 their mean score was significantly higher than those observed at T2 and at T3, respectively (Bonferroni *post hoc* test *p* < 0.005) (Figure 2B).

For the Hearing and Language quotient, results showed significant differences both between and within groups. First, at T2 FT infants had a significantly lower mean score than that reported by VLBW ones, though only in the case of non-anxious mothers (Bonferroni *post hoc* test *p* = 0.017). Considering the differences within groups, ELBW infants showed a mean score at T2 significantly higher than that reported at T3 in both cases of non-anxious and anxious mothers (Bonferroni *post hoc* test *p* = 0.026 and *p* = 0.006, respectively) (Figures 2C,D); moreover, at T2 VLBW infants had mean scores significantly higher than those reported at T1 and at T3 (Bonferroni *post hoc* test *p* = 0.043 and *p* < 0.0001, respectively), though only in the case of non-anxious mothers (Figure 2C).

### Trajectories of GMDS’s Quotients in ELBW, VLBW, and FT Infants as a Function of Time and Maternal Anxiety

In line with the third aim, we explored the trajectories of each GMDS-R quotient in ELBW, VLBW and FT infants as a function of time (model 1) and maternal anxiety (model 2).

#### Locomotor Quotient (A)

**FIGURE 3:**
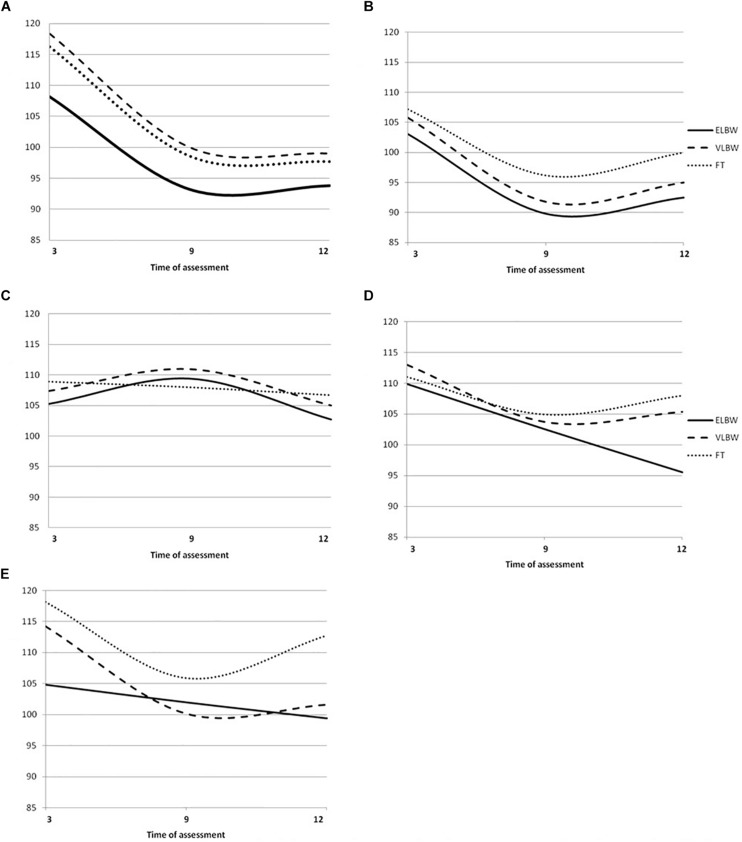
Griffiths Mental Development Scales quotient growth trajectories in ELBW. VLBW and FT infants. **(A)** Locomotor mean Quotients, **(B)** Ferscnal-Social mean Quotients, **(C)** Hearing and Language mean Quotients; **(D)** Eye-Hand Coordination mean Quotients; **(E)** Performance mean Quotients Continue line denotes ELBW group; dashed line VLBW group; dotted line FT group.

In the model 1 (unconditional model), the −2 log likelihood model comparison tests indicated that the average trajectories in ELBW (χ^2^_(__1__)_ = 10.72; *p* < 0.01), VLBW (χ^2^_(__1__)_ = 24.18; *p* < 0.01) and FT infants (χ^2^_(__1__)_ = 34.85; *p* < 0.01) were characterized by a significant negative linear slope, followed by a positive quadratic (curved) slope, indicating a U-shaped pattern (Figure 3).

In the model 2 (conditional model), results showed that in the case of FT infants, maternal anxiety, even if it did not predict change of the intercept, showed a significant main effect on the linear slope: FT infants with non-anxious mothers had a significantly greater negative linear slope than those with anxious mothers. No significant effect emerged on the quadratic curve.

No significant change of the intercept, as well as of the linear and the quadratic slope emerged in the case of VLBW and ELBW infants.

#### Personal and Social Quotient (B)

Model 1 showed that, according to the −2 log likelihood model comparison tests, the average trajectories in ELBW (χ^2^_(__1__)_ = 19.63; *p* < 0.01), VLBW (χ^2^_(__1__)_ = 19.47; *p* < 0.01) and FT (χ^2^_(__1__)_ = 52.77; *p* < 0.01) infants were characterized by a significant negative linear slope and a positive quadratic (curved) slope, indicating a U-shaped pattern (Figure 3).

In the Model 2, results showed no significant change of the intercept and of the linear and the quadratic slopes as a function of maternal anxiety.

#### Hearing and Language Quotient©

According to the −2 log likelihood model comparison tests, model 1 suggested that the average trajectories in ELBW (χ^2^_(__1__)_ = 9.86; *p* < 0.01) and VLBW infants (χ^2^_(__1__)_ = 15.19; *p* < 0.01) were characterized by a significant positive linear slope followed by a negative quadratic (curved) slope, indicating an inverted U-shaped pattern; while the trajectory of FT infants (χ^2^_(__1__)_ = 0.33; *p* < 0.05) was characterized by a non-significant negative linear slope, indicating a linear pattern (Figure 3).

Model 2 showed that maternal anxiety did not predict change of the intercept, as well as of the linear and the quadratic slope.

#### Eye-Hand Co-ordination Quotient (D)

In the model 1, the −2 log likelihood model comparison tests suggested that the average trajectory in ELBW infants (χ^2^_(__1__)_ = 10.67; *p* > 0.05) was best described by a significant negative linear slope, indicating a linear pattern, while those of VLBW (χ^2^_(__1__)_ = 4.08; *p* < 0.05) and FT infants (χ^2^_(__1__)_ = 9.43; *p* < 0.01) were characterized by a significant negative linear slope and a positive quadratic (curved) slope, indicating a U-shaped pattern (Figure 3).

Model 2 showed that maternal anxiety did not predict change of the intercept, as well as of the linear and the quadratic slope.

#### Performance Quotient (E)

Comparing the fit of the models with the −2 log likelihood model comparison tests, model 1 showed that the average trajectory in ELBW infants (χ^2^_(__1__)_ = 0.01; *p* > 0.05) was best described by a non-significant negative linear slope, indicating a linear pattern, while those of VLBW (χ^2^_(__1__)_ = 23.62; *p* < 0.01) and FT infants (χ^2^_(__1__)_ = 58.03; *p* < 0.01) were characterized by a significant negative linear slope and a positive quadratic (curved) slope, indicating a U-shaped pattern (Figure 3).

Model 2 suggested that, in the case of ELBW infants, maternal anxiety predicts change of the intercept, but not of linear or quadratic slope. In particular, the average score for ELBW infants with non-anxious mothers was 107.22; ELBW infants with anxious mothers started significantly lower by −9.94 points (at about 97.28). No significant change emerged in the case of VLBW and FT infants.

## Discussion

This study aimed at assessing preterm infants’ outcomes in different developmental areas (Locomotor, Personal and Social, Hearing and language, Eye-hand Co-Ordination and Performance) during the 1st year of life, exploring the impact of severity of birth weight, also in relation to postnatal maternal anxiety. A further aim was to describe trajectories of these developmental dimensions in ELBW, VLBW and FT infants from 3 to 12 months postpartum. One of the main strengths of this study was to explore the impact of the severity of prematurity on each GMDS-R quotient in order to highlight possible areas of vulnerability in specific phases of development.

Different results emerged in relation to specific developmental areas, as measured by GMDS-R quotients.

Regarding the Performance quotient, which measures skills in manipulation, speed of working and precision, as well as the ability to apply them in novel situations (Griffith, 1996), a first result showed that, independently from time of assessment, both ELBW and VLBW infants had lower scores than FT ones. However, when the time of assessment was considered in line with the objectives of our study, results showed differences between the 2 preterm groups. At 3 months, ELBW infants had lower scores than VLBW and FT ones, while at 12 months VLBW infants had lower scores than FT ones. At 12 months, the mean scores of ELBW and VLBW infants were quite similar (99.49 vs. 98.02), suggesting that ELBW infants also had a worse outcome, even if not statistically significant, compared to FT infants. These findings seem to suggest that, for ELBW infants, difficulties related to performance domain arise early, at 3 months (having quite stable mean scores across time). For VLBW infants, whose score significantly decreased from 3 to 9 months postpartum, difficulties would arise later (around 12 months).

The presence of maternal anxiety did not seem to have a significant impact on infants’ mean scores of performance quotient, independently from the birth weight classification.

Furthermore, growth trajectories analyses underline that ELBW infants showed a non-significant negative linear pattern of growth, while VLBW and FT infants demonstrated a U-shaped pattern of growth. These findings suggest that, during the 1st year postpartum, VLBW and FT infants had a similar trend of development, even if VLBW infants showed lower scores across time. The only significant result in the case of maternal anxiety emerged on the intercept of ELBW infants: ELBW with anxious mothers showed significantly lower scores than those without anxious mothers.

The decrease of quotients observed in the study is somehow unexpected. However, a possible explanation could be given by the increase of the complexity of task demands required by the GMDS-R (Griffith, 1996). Indeed, in the 1st months, very simple and general abilities are required of the infants, while in the following months more complex tests are provided, requiring the skills to respond to items of increasing difficulty and to unusual stimuli. Thus, it could be possible that performance of ELBW infants, due to the severity of their condition, could be influenced since the first assessment. Despite VLBW babies not showing difficulties at 3 months, lower scores emerged at 12 months, when the items (put block in a box, use of form-boards, etc.) required precision, adaptability and a capacity to persist in a task; these abilities are complex and may still not be fully acquired, as in the case of FT infants.

These results may suggest that the development of ELBW and VLBW infants could benefit from *ad hoc* interventions; in particular, in the case of ELBW babies, interventions aimed at promoting very simple and general abilities should start since the first postpartum months of life, while in the case of VLBW babies, the therapeutic interventions, aimed at building more complex abilities, could start later.

To our knowledge, no previous studies have explored ELBW, VLBW and FT infants’ performance development across time, nor their trajectories of growth, by also considering the role of maternal anxiety. Therefore, further studies are recommended.

Regarding the Hearing and Language quotient, it is relevant to note that most of the infants did not present an index of delay, defined as a score < 1 DS. This result is unexpected and is not in line with previous studies (Cattani et al., 2010; Sansavini et al., 2011; Ballantyne et al., 2016; Ionio et al., 2016; Cheong et al., 2017; de Jong et al., 2017; Lean et al., 2018; Pisoni et al., 2018), in which preterm infants have shown worse linguistic development than FT ones. A possible explanation could regard the time of assessment: all the previously mentioned studies mainly focused on the 2nd year postpartum, while the present study focused on the 1st year postpartum. Thus, it could be possible that the impairments described in previous studies are not yet detected during the 1st year of life. However, in order to identify possible early signs during the first postpartum months of infant age, some considerations about results on continuous scores could be given.

A first consideration is about the significant decrease in scores from 9 to 12 months shown by ELBW infants. This effect emerged both in the case of anxious and non-anxious groups. Conversely, we observed this decrease in VLBW only if their mothers were non-anxious and it never emerged for FT infants. These results could be explained considering the steps of the acquisition of hearing and language skills during the 1st year: the period between 9 and 12 months is a sensitive and pivotal time in which a baby should show more complex linguistic abilities, such as using gestures (e.g., waving and pointing) and vocalizations (e.g., “mummum,” “dada,” and “tete”). For this reason, during the first postpartum months of an infant’s age, items of GMDS-R scales are mainly focused on hearing skills, while at the end of the 1st year the quantity and quality of infant vocalizations are deeply assessed. So, the decrease in the scores from 9 to 12 months could show how the adaptation to new skills is highly demanding in the case of preterm infants, especially for high-risk babies like ELBW ones, as we found in a previous research (Neri et al., 2017). This trend for preterm infants is also shown by the trajectories analysis, where ELBW and VLBW infants showed an inverted U-shaped pattern of growth, and supported by Greene et al. (2013), who found that preterm infants at 8 months obtained lower scores in expressive than in receptive language.

Conversely, full-term infants might have already found an adjustment on these abilities, thus showing more stability in their scores throughout all the assessments.

At 9 months postpartum, VLBW infants showed a higher quotient compared to the FT group and this result emerged only in the case of non-anxious mothers. This somehow unexpected result may possibly be explained considering the fact that the VLBW group represents a low risk sample and it could have taken advantage of the supportive interventions realized in NICU and during the follow-up programs (Biasini et al., 2015; Montirosso et al., 2016; Neri et al., 2017), aimed at promoting both infants’ communicative skills and parents’ ability to support infant vocalizations. However, when maternal anxiety is present in the VLBW group, this may mediate the efficacy of interventions in improving Hearing and Language skills.

Further studies including the role of maternal anxiety are recommended.

To sum up, our results suggest that more evident difficulties for hearing and language development manifest at the end of the 1st year postpartum, when a baby should start to use gestures, vocalizations and single words to communicate. These findings are important because they highlight the importance of planning early language-focused interventions in order to limit these subsequent difficulties (Stolt et al., 2016).

Regarding the Locomotor quotient, ELBW, VLBW and FT babies had similar scores, independently from time of assessment. This result is not in line with previous studies, highlighting that preterm infants, compared to FT ones, had significantly more difficulties in acquiring gross motor skills in the 1st and 2nd year of life (Mukhopadhyay et al., 2010; Fallah et al., 2011; de Souza and de Castro Magalhães, 2012; Ballantyne et al., 2016; Cheong et al., 2017; de Jong et al., 2017; Lean et al., 2018). Regardless, none of these studies differentiated preterm infants in relation to the severity of prematurity. Only 2 studies compared 3 gestational age groups (Sansavini et al., 2010) or 3 birth weight groups (Neri et al., 2017), showing, in the first case, significantly lower locomotor scores in ELGA infants compared to VLGA and FT infants; in the second study, no significant differences among ELBW, VLBW and FT samples were reported. Further studies are needed to better describe whether the severity of prematurity impacts on locomotor development differently.

When maternal symptomatology was also included, specific patterns relating to anxious and non-anxious groups emerged. When mothers were non-anxious, the mean scores observed in ELBW, VLBW and FT infants significantly decreased from 3 to 9 months, followed by relatively stable scores from 9 to 12 months. In this case, the Locomotor quotient tends to show a specific trend of development across time, independently from the severity of birth weight.

Conversely, when mothers were anxious, ELBW and FT infants showed similar scores from 3 to 12 months postpartum, while only VLBW infants maintained the decrease observed from 3 to 9 and 12 months. It is unexpected for VLBW infants to reach good performance at T1 despite maternal anxiety. Previous literature has suggested that, in the case of preterm birth, anxiety could function as an adaptive response to traumatic condition (Neri et al., 2015), helping mothers to maintain the focus on the infant. It could be possible that the adaptive role played by anxiety could emerge, especially in the case of VLBW infants, most likely due to their less severe condition of prematurity, as well as to the supportive intervention offered by the NICU.

This result confirms the way in which ELBW and VLBW may show different profiles in the acquisition of Locomotor skills, with different time of improvement and different resources to environmental stimulations.

To our knowledge, no previous studies have explored the differences between ELBW, VLBW and FT infants on locomotor development at 3, at 9 and at 12 months of life, considering the presence of maternal anxiety and this study represents a first step in this direction.

Lastly, considering Eye-hand Co-Ordination and Personal and Social quotients, significant results did not emerge according to severity of birth weight and maternal anxiety.

In the case of the Eye-hand Co-Ordination quotient, ELBW infants presented a small prevalence of delays in all the assessments and a negative linear pattern, different from the U-shaped pattern reported by VLBW and FT infants. This result is in line with previous studies (Greene et al., 2013; Lobo et al., 2014), reporting that few preterm infants had impairment in fine motor skills and that this may reflect a tendency to worsen across time. Specifically, Greene et al. (2013) showed an impairment on fine motor skills in the 2nd year of life in a sample of premature infants. Therefore, the negative linear pattern of growth shown by ELBW infants in Eye-hand Co-Ordination may underline that ELBW infants could obtain low scores that will became impairment in the second year of life, as emerged for Greene et al. (2013). It is interesting to evidence that Locomotor and Eye-hand Co-Ordination quotients give a measure of the quality of infant motor abilities (Griffith, 1970, 1984). However, while the Locomotor scale assesses gross motor skills, including the ability to balance and to co-ordinate and control movements, the Eye-hand Coordination Subscale evaluates fine motor skills, manual dexterity and visual perceptual skills. Future studies should compare ELBW, VLBW and FT infants on locomotor development across the 1st year postpartum, differentiating the development of gross motor and fine motor skills.

In the case of the Personal and Social quotient, no significant differences emerged when parametric analyses were run; nevertheless, relevant changes were observed in the rate of delay in the 3 groups. In particular, during the assessment at T2, 9 months, a very high rate of delay was present in the preterm group, especially in the case of ELBW infants (44%). Nine months represent a sensitive period for development, as babies more actively interact with the surrounding environment and new skills are learned, especially in the food area; e.g., the consolidation of weaning leads to increasing autonomous behaviors, like taking foods by hands, attempting to drink from the bottles alone, etc. The acquisition of these skills could be more difficult for severely preterm infants and for their parents; indeed, memories of previous experiences during hospitalizations (apneas, difficulties on breast-feeding and breathing) could interfere with the scaffolding role that parents could play. Therefore, the clinicians need to pay specific attention to the meaning that feeding has for these families. Furthermore, results of growth curve analysis showed that, despite not significant, preterm infants obtained lower mean scores than FT ones, with a U-shaped pattern.

A relevant consideration is required about the use of developmental scales for the assessment of relational skills, rather than the evaluation by interactive scales, as in previous literature (Korja et al., 2012; Agostini et al., 2014; Bilgin and Wolke, 2015). As suggested in previous studies, it could represent a bias (Lobo and Galloway, 2013; Lobo et al., 2014). Further studies should consider the possible correlations between the results found by these different instruments of assessment.

To our knowledge, this is the first study that longitudinally explored the impact of the severity of birth weight along with maternal anxiety on each GMDS-R quotient, in order to understand whether specific areas of development are more exposed to impairments across the 1st year postpartum.

Taken together, the results may suggest a discrete instability of the scores at 3-, 9-, and 12-months, as assessed by GMDS-R, strengthening the evidence from previous research that the assessment of infant development by developmental scales would show lower sensitivity in the case of high risk infants (Janssen et al., 2011; Greene et al., 2013; Lobo and Galloway, 2013), compared to infants with typical development. The instability of the assessment of high-risk infants, like preterm ones, could represent a limit of developmental scales (GMDS-R or Bayley) and suggests the need of a different kind of evaluation during follow up in the 1st year of infant life (Lobo et al., 2014).

Though this may suggest a limit regarding the methodology of the study, some clinical implications for intervention may arise. First, the assessment of high-risk infants and first signs of delay in the context of preterm birth should benefit from the inclusion of a series of diagnostic and observative instruments (e.g., observation of infant during free play; see Lobo et al. (2014). Second, fluctuations of scores in GMDS-R dimensions may suggest that, during the 1st year of life, there are several sensitive periods for the different developmental areas. Therefore, the transition across the 1st year of life may be challenging. Thus, during a follow up program, parents should be supported to read infant cues and to provide them with the most adequate learning experiences possible (Lobo and Galloway, 2013).

Several limits of the study may be acknowledged. First, the results need to be confirmed on wider samples. In particular, our ELBW and VLBW samples are smaller than the FT ones; this difference could have influenced the detection of differences among the 3 groups.

Second, regarding maternal anxiety, we chose to focus on worries, a specific component of generalized anxiety, as preterm mothers may tend to worry excessively about infant health long after discharge from the hospital. However, it may be possible that other components of anxiety emerge during the 1st year in the context of a preterm birth, such as post-traumatic symptoms or generalized anxiety, as suggested by previous studies (Correia and Linhares, 2007; Padovani et al., 2008), therefore they would need to be measured. This could in part explain why we did not find a relevant influence of anxiety on infant development compared to previous studies (Zelkowitz et al., 2008, 2009, 2011; Glasheen et al., 2010; Keim et al., 2011). Besides, the choice to assess maternal symptomatology only at 3 months may have influenced our results.

Third, we did not investigate the effect of maternal depressive symptoms, which often occur in comorbidity with anxious symptomatology (Garfield et al., 2014; O’ Hara and Wisner, 2014; Yang et al., 2017), and we did not assess the quality of mother-child relationships that, in the case of anxiety, may interfere with caregiving practices (Beebe et al., 2011; Pisoni et al., 2018), representing a risk factor for infant development. Further studies should also include these factors.

Besides, for a more accurate understanding of the results, it is worth noting that preterm dyads were recruited in a NICU, where all procedures are based on Developmental Care (Vandenberg, 2007) and the staff demonstrate a high level of expertise in protecting and enhancing the infant’s and parents’ quality of life. During hospitalization, parents have a 24-h free access to the Unit and their abilities to recognize and to adequately respond to infants’ cues are constantly supported. Furthermore, after discharge, the families are included in a follow-up program, where both infant development and parental affective state are monitored. All these variables need to be considered for their possible influences on the results of the study.

Globally, the results suggest that the severity of birth weight, also in possible interaction with specific aspects of maternal anxiety (tendency to worry), have significant impact on infant development across the first postpartum year.

For this reason, the categorization based on severity of birth weight should always be considered when the impact of a preterm birth on child development is investigated; along with this, specific attention should be paid to different developmental dimensions and their trajectories, in order to underline possible infant vulnerabilities and strengths. Specifically, *ad hoc* tailored interventions should be promoted to assess the risk of preterm infants’ delay and anxiety symptoms with adequate tools, to offer special support and treatment for symptomatology and to enhance parental functioning. This could help to implement more accurate interventions, as suggested by Developmental Care (Burke, 2018).

## Data Availability Statement

The datasets generated for this study are available on request to the corresponding author.

## Ethics Statement

Ethical approval for this study was given by the Department of Psychology (University of Bologna, Italy). Written informed consent to participate in this study was provided by the participants’ legal guardian/next of kin.

## Author Contributions

EN prepared the study design, organized the sample recruitment, collected data, performed statistical analysis, and contributed to the writing of all the sections of the manuscript. FG performed statistical analysis and contributed to the writing of all the sections of the manuscript. FM, ET, AB, and MS contributed to the preparation of the study design and supervised data collection and the research team. FA prepared the study design, supervised all the phases of the research study and contributed to the writing of all the sections of the manuscript. All authors reviewed and approved the manuscript for publication.

## Conflict of Interest

The authors declare that the research was conducted in the absence of any commercial or financial relationships that could be construed as a potential conflict of interest.
